# Circular economy and 3D printing in the healthcare sector

**DOI:** 10.3389/fbioe.2025.1548550

**Published:** 2025-03-27

**Authors:** Nada Khaled Mansour, Arianna Callera, Federica Potere, Simone Micalizzi, Maria Laura Costantino, Francesco De Gaetano, Paolo Oliva

**Affiliations:** ^1^ IRCCS Humanitas Research Hospital, Milan, Italy; ^2^ Department of Chemistry, Materials and Chemical Engineering “Giulio Natta”, Politecnico di Milano, Milan, Italy

**Keywords:** 3D printing, circular economy, recycling, healthcare, high-density polyethylene, fused deposition modeling

## Abstract

**Introduction:**

In the last decades, 3D printing has demonstrated its potential across various sectors, including healthcare. However, there remains a notable gap in the integration of circular economy principles to recycle plastic waste into functional, high-quality 3D printing filaments, particularly in clinical settings. This work addresses this gap by exploring the sustainability of 3D printing in healthcare through the recycling of plastic waste into 3D printable filaments.

**Methods:**

The process involves the collection, shredding, extrusion, and spooling of high-density polyethylene (HDPE) water bottle caps, collected from hospital setting. Key steps, such as extrusion and printing processes, were optimised, and the mechanical properties of the filament were thoroughly assessed. An economic and an environmental impact analysis was also conducted to evaluate the overall process. Optimization of each phase of the circular economy process led to the production of a functional recycled filament, with homogeneous diameter and surface finish quality.

**Results:**

Despite HDPE being challenging to print, targeted adjustments significantly enhanced the print quality. The study not only aimed to obtain a usable filament but also to assess the economic and environmental impact of the whole process. The results indicated cost saving from in-house filament production compared to commercial options and a notable reduction in the environmental impact measured in carbon dioxide (CO_2_) emission equivalent. The recycled filament was successfully used to print a patient-specific anatomical model of an intracranial aneurysm, as a support for surgical planning.

**Discussion:**

This demonstrates the feasibility of integrating sustainable 3D printing practices in healthcare, offering economics and environmental benefits while enhancing clinical support.

## 1 Introduction

Additive manufacturing technologies represent a powerful tool able to develop a variety of objects, making it exploitable in several sectors. In healthcare, 3D printing is increasingly utilized due to its rapid production capabilities and ease of use. One of the main applications of 3D printing in hospital is the production of patient-specific anatomical models, which can assist in surgical procedure. These 3D reproductions of the region of interest enable surgeons to better identify the surgical strategy to adopt, leading to improved clinical outcomes and reduced operative times. Moreover, for complex procedures, patient-specific medical devices, such as 3D printed surgical guides, can be produce to assist surgeons during operations (Aimar et al., 2019). Additionally, patient-specific 3D printing models facilitate the explanation of therapies and procedures to patients aiding in the development of a trust-based relationship. This approach can help contrast the increasing skepticism towards healthcare providers.

High-density Polyethylene (HDPE) is a thermoplastic polymer derived from petroleum with a generalized chemical formula (C_2_H_4_)n, representing the repeating ethylene monomer unit that constitutes the polymeric structure of the plastic material. HDPE can be found in high concentration in hospital settings thanks to its low moisture absorption, chemical resistance, recyclability, and low bacterial retention rate (Graziano et al., 2019).

Moreover, HDPE is commercially available as 3D printing filament, indicating its potential for applications in additive manufacturing. However, its limited use by consumers highlights the challenges in the printing process, mainly related difficulties in adhesion to the printing plate and significant shrinkage. Despite these drawbacks related to HDPE, it remains a valuable material for the healthcare sector. In addition, the printing challenges can be overcome by tuning the printing parameters.

Plastic materials are widely utilized in healthcare due to their versatile mechanical properties, low weight, and cost-effectiveness. The plastic production process causes environmental harm due to the production of carbon dioxide, a potent greenhouse gas contributes to global warming by absorbing infrared radiation. Moreover, plastics are estimated to account for about 30% of all healthcare waste (Rizan et al., 2020; Kenny and Priyadarshini, 2021; ISPRA, 2022).

The concept of circular economy has evolved over time, with several definitions, emphasizing the efficient use of resources, the adoption of renewable energy and the reduction of waste (Rizos et al., 2017; Geisendorf and Pietrulla, 2018; Korhonen et al., 2018). In this study, the term “circular economy” aligns with the European Environmental Agency’s 2014 definition, which underlines that circular economy “focuses on recycling, limiting and re-using the physical inputs to the economy, and using waste as a resource leading to reduced primary resource consumption”. (Ekins et al., 2019).

Given the environmental impact of healthcare waste and the promising potential of 3D printing in the medical field, this research explores the feasibility of implementing a circular economy process to recycle HDPE plastic bottle caps into 3D printable filament.

## 2 Materials and methods

At Istituto Clinico Humanitas (ICH), in Milan, Italy, a process was designed to recycle non-hazardous healthcare plastic waste into 3D printable filament, with careful consideration of each step and its specific requirements. Given the wide range of plastics used in the healthcare, the proof of concept initially focused on a single type of plastic: HDPE.

This choice was based on two key factors: the widespread availability of HDPE, particularly as the primary material for bottle caps; and its favorable mechanical, chemical and thermal properties (Cuadri and Martín-Alfonso, 2017), which make it suitable for various applications, especially in the healthcare sector. The efficiency of the recycling process was assessed based on several criteria, including the mechanical properties of the resulting filament, its economic impact on the hospital, environmental sustainability, and its printability. Finally, a case study was conducted to validate the process.

### 2.1 Workflow

**FIGURE 1:**
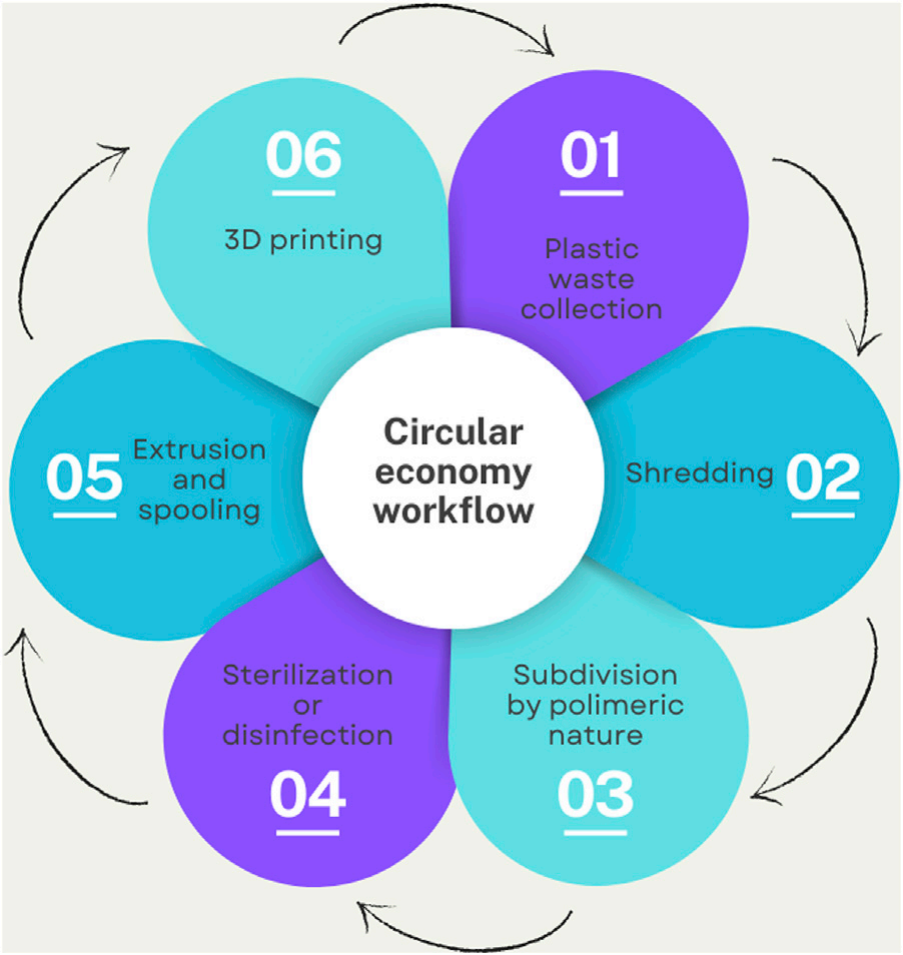
Theoretical circular economy workflow. Step 1: plastic waste collection, step 2: shredding into homogeneous ground, step 3: subdivision of shredded material based on polymeric nature, step 4: sterilization or disinfection, step 5: extrusion of ground material into printable filament and lastly step 6: 3D printing.

The process involves six different steps, that considered the complexity of creating a filament starting from various plastic materials, with very different thermal and mechanical properties. The workflow is shown in Figure 1.

The first step consists on the collection of plastic waste. This requires the implementation of a system that effectively sorts waste, not only separating glass, plastic, and paper - as is already practiced at ICH - but also introducing specific containers for specific types of plastic. This approach would enable the correct reprocessing of materials to produce single-polymer filaments. However, this step represents a significant challenge, as it requires a changes in behaviour among operators who must take extra care to sort plastics according to labelled categories. Moreover, medical devices consist of multiple types of plastics, complicating the labeling and proper disposal process (Groh et al., 2019). Therefore, an automated sorting process is proposed in subsequent steps as an alternative to manual sorting.

The second step is the shredding of the collected plastic waste. This step yields a uniformly ground material. At this point sorting by polymer nature becomes necessary in order to correctly recycle the different plastics.

Therefore, the third step of the workflow is subdivision, obtainable through automated sorting technologies, such as X-ray technology (XRF and XRD). These technologies able to detect heavy elements such as chlorine and bromine, differentiating PVC from look-alike PET, since both plastics are transparent and used for packaging (Turner and Filella, 2017; Shaw and Turner, 2019). Accurate material separation ensures that appropriate sterilization methods can be selected, tailored to the specific properties of each plastic type.

The fourth step is the sterilization of the sorted plastic waste, aimed at reducing bacterial contamination to acceptable levels. Although the healthcare waste considered in this process is non-hazardous, the risk of contamination remains elevated in hospital environments, necessitating effective sterilization. The choice of sterilization method depends on the initial level of contamination as well as the thermal and mechanical properties of the materials, ensuring that these properties are preserved throughout the process.

The fifth stage is the extrusion, where the sorted and sterilized material is melted. Temperature and extrusion speed are carefully controlled to produce filament suitable for 3D printing. The final filament is then used in 3D printing, and any support structures or failed prints are reprocessed to obtain new filaments, thereby closing the recycling loop.

**FIGURE 2:**
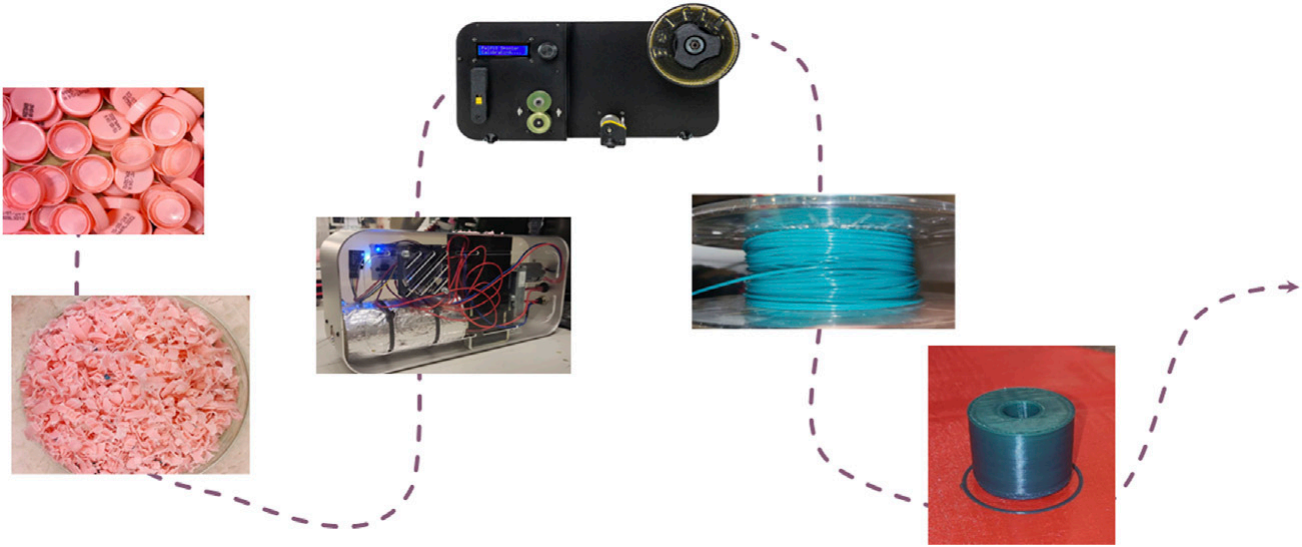
Actual workflow implemented. Step 1: plastic bottle caps collection, step 2: shredding into homogeneous ground, step 3: extrusion of ground material into printable filament, step 4: spooling of recycled filament and step 5: 3D printing.

We outline the final workflow of implementing a circular economy process for the recycling HDPE within a hospital setting. The two main differences from the initial workflow for recycling plastic healthcare waste are the absence of the division process based on polymeric nature, as all collected plastic is made of the same material, and the omission of the sterilization step, as the bottle caps were collected before disposal and extrusion occurred at a temperature sufficient for disinfection. However, this study did not conduct specific analyses on the bacterial load. The steps of the implemented workflow are shown in Figure 2.

#### 2.1.1 Bottle caps collection

**FIGURE 3:**
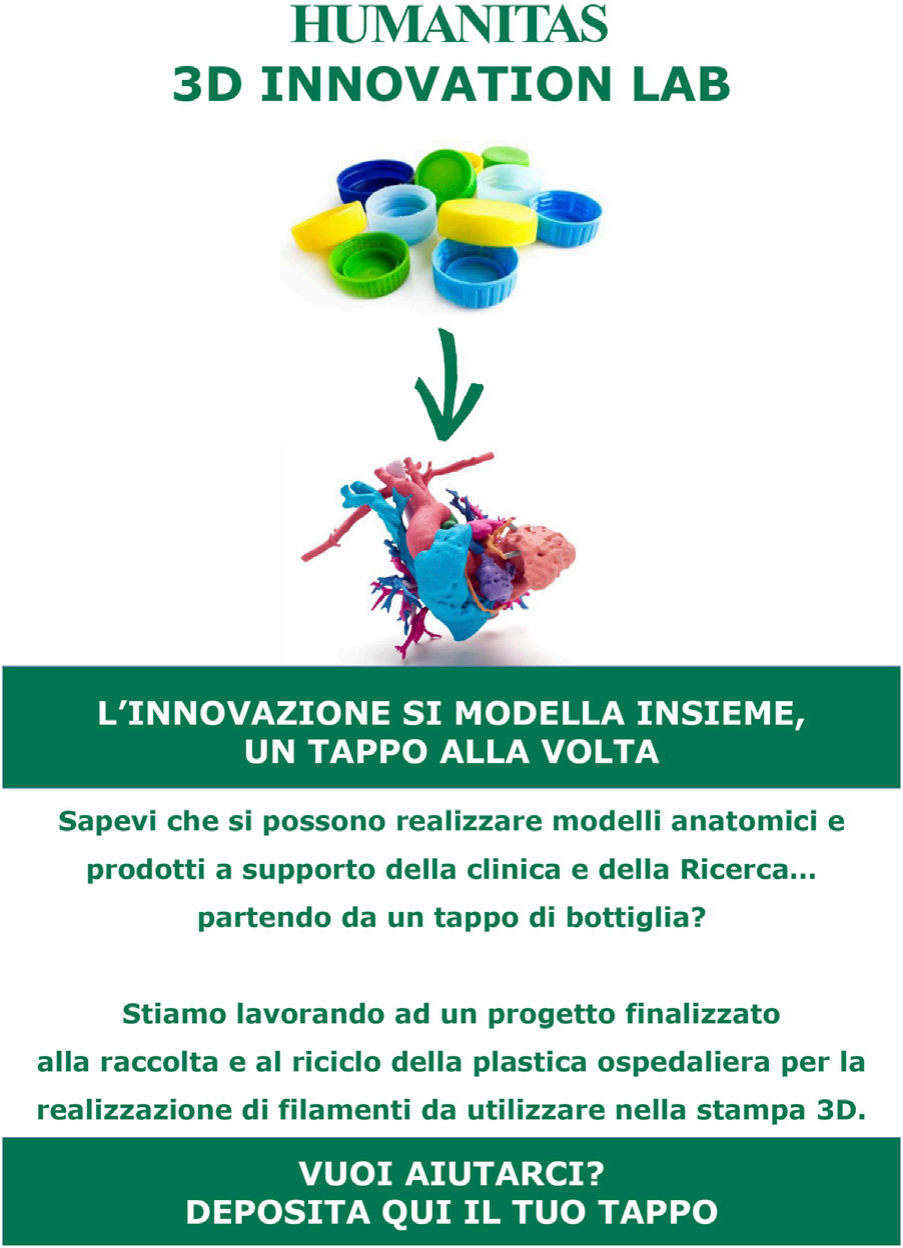
Leaflet from communication team to enhance engagement into bottle caps collection.

The collection of plastic bottle caps was promoted by the communication team of ICH, that realized a leaflet outlining the project’s goals and the potential benefits (Figure 3), to encourage operators’ participation. A pilot trial was launched in a cardiology recovery ward, where a 30 × 40 × 30 cm collection box was introduced on the 1 September 2023, to gather bottle caps.

To further motivate the operators’ involvement, a 3D-printed anatomical heart was printed to show them the potential use of the bottle caps they were collecting.

#### 2.1.2 Subdivision

Different colours were identified with the aim of creating coloured filaments: blue, pink and green filaments were produced, along with transparent and mixed ones.

#### 2.1.3 Shredding

The shredding phase is essential to obtain a homogenous ground plastic material. The key parameter to tune is the number of shredding cycles necessary to reach the desired dimension for the grounded material. Felfil Shredder (*Felfil 750, Turin, Italy*) was used for this purpose. Additionally, it is important for the individual plastic pieces to have similar dimension to avoid issues in subsequent processing stages. To ensure homogeneity a 3D printed sieve was used, with an average diameter of 8 mm.

#### 2.1.4 Extrusion

The extrusion phase represents a crucial point in the process, considering how the quality of the resulting filament influence the printing potential.

Even though the nozzle of the extruder has a diameter of 1.75mm, the resulting filament can have a non-constant diameter, resulting in an unusable filament for 3D printing. Moreover, the porosity of the resulting product can additionally influence the printing potentialities of the filament since air bubbles can cause an intermittent emission of material from the printer nozzle.

Felfil Evo Extruder (*Felfil Evo complete kit, Turin, Italy*) and Felfil Spooler+ (*Felfil Spooler+, Turin, Italy*) were used for this purpose. The extruder presents a K100 steel screw with an L/D ratio of 12.7. For the purpose of this study, all equipment is not required to be pharma grade, since the printed product do not come in contact with patients. Additionally, HDPE does not require water cooling, therefore the air cooling system included in the Felfil Spooler+ was sufficient.

**FIGURE 4:**
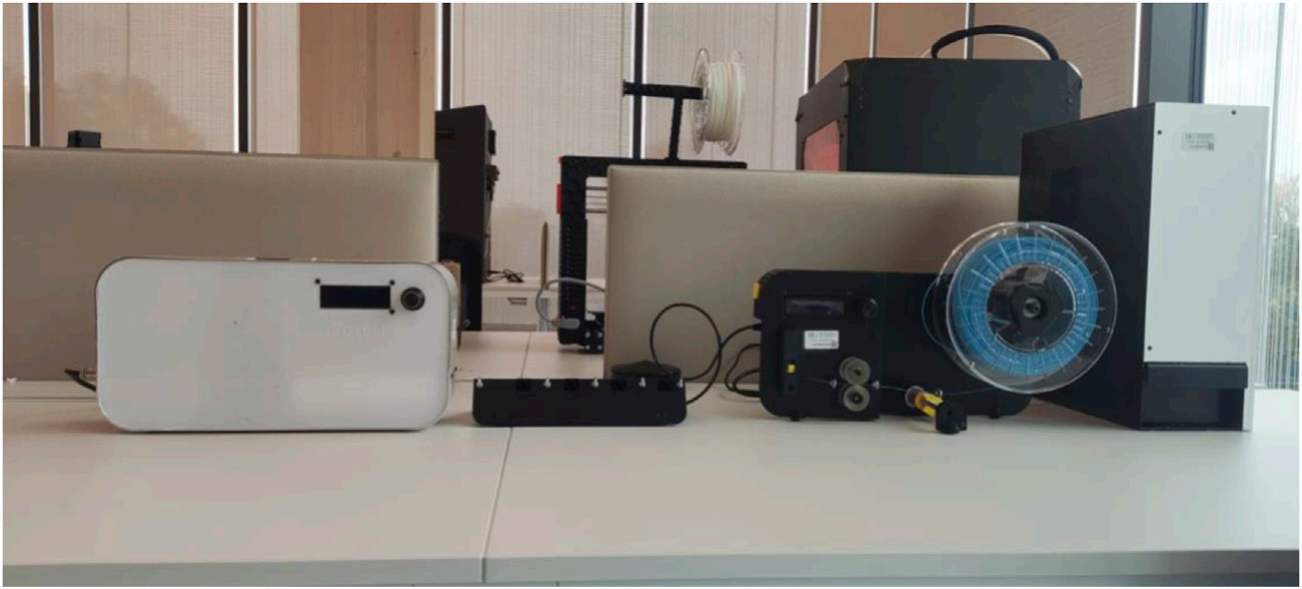
Extrusion and spooling process. From left to right: extruder, cooling system and spooler.

The extrusion and spooling process is shown in Figure 4.

#### 2.1.5 Printing

3D printing encompasses a laundry list of parameters to tune, that can vary a lot based on the chosen material. For HDPE, since it is not a very common material to print, information about the optimal values is scarce. The publication “3D printing of high-density polyethylene by fused filament fabrication” by *Schrimeister et al.* was used as a starting point, using the same printing temperature, the build plate temperature and the printing speed, as indicated in Table 1 (Schirmeister et al., 2019). The parameters were then adjusted based on the quality of the resulting print, focusing in particular on adhesion, regularity of printing and ease of detachment of the support structure. A 3D printer Kentstrapper Verve (*Kentstrapper, Florence, Italy*) was used for this purpose.

**TABLE 1:** Printing parameters for HDPE. In particular, wide ranges were used as starting point.

Parameter	Value
Nozzle Temperature [°C]	240–260
Build Plate Temperature [°C]	60
Printing Speed [mm/s]	25–150

### 2.2 Mechanical characterization

Mechanical properties were analysed using electromechanical testing machine (MTS Synergie 200H, Eden Prairie, MN) equipped with a 100 N or 1 kN load cell, evaluating different set-ups through tensile tests. The considered parameters for the evaluation were Young’s modulus and Yield stress, both obtainable from the stress-strain diagram. The values were then compared to the values of commercially available HDPE filament, specified in the data-sheet.

#### 2.2.1 Test 1

**FIGURE 5:**
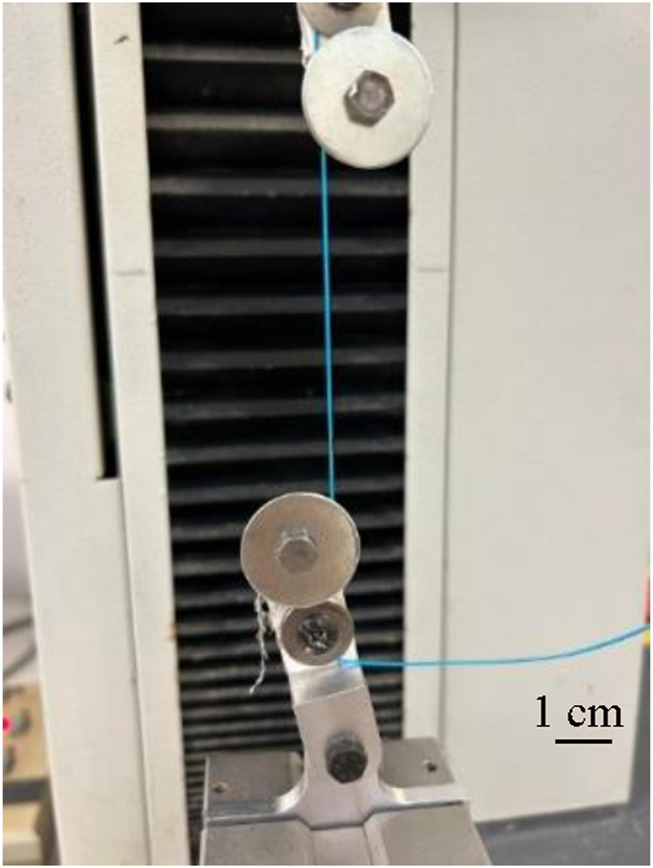
Traction set-up for thin filament. The filament was spooled on the clamps in order to reduce tension outside of the test length.

The first test was performed using a thin filament, with an average diameter of 0.5 mm. The set-up consisted of two coils and two attachment screw, as shown in Figure 5 and well described by Pagnanelli et al. (2023). The aim behind the design of the set-up used is to decrease the stress on the filament due to the attachment to the structure, allowing the behavioural study in the test length. For this reason, the filament is guided around the coils. Moreover, the results emerging from this first test are independent of the printing process, allowing a more precise characterization of the produced filament.

The parameters used for the first test were chosen based on the characteristics of the filament and the expected values of break point. Considering the small diameter, a load cell of 100 N was used. The set speed was 0.1 mm/s, since the test length imposed is around 5 cm.

#### 2.2.2 Test 2

The second test was conducted with the same set-up described in *Test 1*, using a thicker filament. The nominal diameter of the filament tested was 1.5mm, closer to the diameter accepted by most 3D printers. The test length was 6.5cm, while the other parameters were set equal to *Test 1*.

#### 2.2.3 Test 3

**FIGURE 6:**
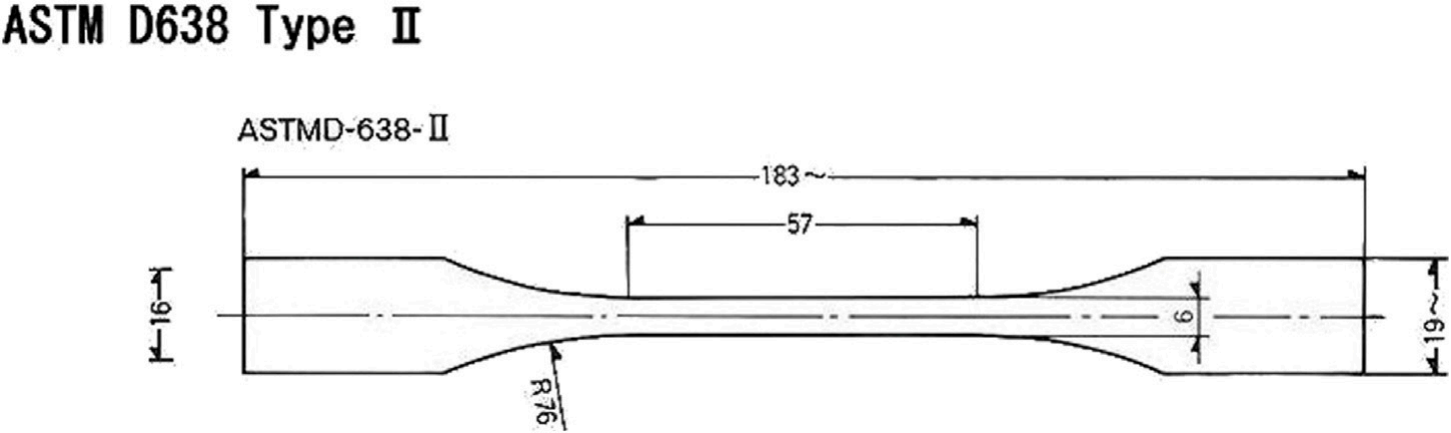
ASTM specimen type II.

Test 3 was conducted on a Type II dog-bone specimen according to ASTM D638 Standard, which measurements are shown in Figure 6. The specimen was designed using FreeCAD (FreeCAD). Firstly, a 2D sketch was designed, following the measurements in Figure 6, then an extrusion operation was performed.

The test length was 5.7 cm, the load cell was 1 kN and the set speed was 0.2 mm/s.

The aim of this test, beyond characterising the mechanical properties of the material, is to evaluate the impact of the printing process. Specifically, the specimen was printed in order to have vertical layers, oriented in the same direction of the applied force (Rodrigues et al., 2023).

### 2.3 Economic sustainability evaluation

The economic analysis was conducted by evaluating the impact of each phase of the process on the overall cost balance. Four key areas of interest were identified: equipment costs (including purchasing and maintenance costs, amortised over the hypothesised life of the machines), energy consumption costs (measured in euro (€) per kWh), raw materials costs and labour costs. The adjusted values for these factors have been then compared to the costs per kilogram of an industrially produced HDPE filament. Four distributors were selected for the analysis: 3DStore Monza, Filoprint, Digitec and Stampa3DSud, all of them located in Italy or Switzerland.

For this analysis, hourly amortised costs were estimated, assuming an average machine lifespan of 8 years. Therefore, the economic impact of the equipment was calculated using the following equation:

$$C_{eq}\left(x,m\right)=\frac{\left(\frac{x}{8}\right)+m}{2080}$$

where *x* is the price of the purchased equipment [€], *m* is the yearly maintenance cost [€], and 2080 h/yr and the estimated working hours in a year.

The energy consumption is calculated as follows:

$$C_{en}\left(r,y\right)=\left(\frac{1}{r}\right)\cdot y\cdot 0.0625$$

where *r* is the amount of material processed in 1 h [kg/h], *y* is the average consumption of the equipment [kWh], and 0.0625 €/kW is cost of 1 kW on energy consumption.

Since the costs of raw material was considered to be negligible, the man labour cost were evaluated with the following equation:

$$C_{ml}\left(t\right)=t\cdot 7$$

Where *t* [h] is the time required for the processing of 1 kg of material and 7 €/h is the average wage for man labour in Italy.

For the term of comparison, the mean value of the cost per kilogram of commercially available HDPE filament was considered. In particular, a 30% discount was applied on the list price and then normalized for 1 kg of material.

### 2.4 Environmental sustainability evaluation

An environmental sustainability analysis was performed to compare the CO_2_ emissions of the implemented circular economy process and the industrial process. Three key aspects contributing to the CO_2_ emission were considered: raw material extraction, filament production and transportation. For what concerns industrial products, the same four distributors were analysed. The limited transportation distance was selected to reflect moderated transportation time and distance, minimizing the possible environmental impact.

Regarding raw materials extraction, this phase was only evaluated for the industrial process, as the material in the presented project is fully recycled. For the extraction of 1 kg of HDPE, 1.75 kg of petroleum is needed. HDPE is considered one of the plastics that has the lower environmental impact, due to its straightforward production process, thus the estimated production of carbon dioxide for the extraction and the refinement of 1 kg of HDPE is 3.11 kg/CO2 (Ram Bhusal et al., 2021).

On average, the production of 1 kg of HDPE filament for 3D printing can generate between 6 kg and 13 kg of equivalent emission, considering all the associated processes. The value considered for this analysis is 9.5 kg.

Following the energetic consumption estimated to produce 1 kg of filament shown in the economic analysis, the amount of carbon dioxide was calculated, knowing that 1  MW h of consumption produces 0.483 tons of CO2.

The equation used is the following:

$$C_{en}\left(r,y\right)=\left(\frac{1}{r}\right)\cdot y\cdot 0.483$$

where *r* is the amount of material processed in 1 h [kg/h] and *y* is the average consumption of the equipment [MWh].

### 2.5 Filament printability

The parameters specified in Table 1 were used as a starting point.

Subsequently, the above parameters were tuned in order to optimize the printing process, considering three main aspects: adhesion to the build plate, warping during and after printing and layer-to-layer adhesion.

### 2.6 Case study

A 32-years old patient was diagnosed with intracranial aneurysm (IA) during a routine check-up. Due to unclear positioning and orientation of the aneurysm in the clinical images, the surgeon requested a 3D model to better visualize the aneurysm and plan the most appropriate surgical approach (Zhao et al., 2018).

In this case study, a Magnetic Resonance Image (MRI) and a MRI with contrast agent were performed. However, the two-dimensional clinical images were insufficient for a clearly visualization of the aneurysm structure, making it difficult to plan the surgery. Therefore, a 3D model was requested. The MRI with contrast agent, which was provided directly by the surgeon, offered better resolution and allowed for clearer distinction of the aneurysm compared to a standard MRI, enhancing the quality of the 3D model. The segmentation process was carried out with the assistance of the surgeon, who regulated the contrast and gave indications on the region to be segmented. The segmentation software used was ITKSnap (ITKSnap, United States, Utah), and no additional refinement was necessary, in accordance with the surgeon feedback.

The obtained 3D model was then processed through PrusaSlicer (Prusa Research a. s, Czech Republic), a slicing software that converted the mesh model into printing instruction, and printed using the recycled filament, with the printing parameters identified in the optimization process of the printing phase.

## 3 Results

### 3.1 Extrusion process

The plastic caps were shredded four times to prepare the material. The spooler was employed to enhance the homogeneity of the filament, specifically focusing on achieving a consistent diameter of 1.75 mm. The manual mode was used, with adjustments made to all parameters until the desired result was achieved.

The final parameters selected are summarized in Table 2. These values correspond to the initial condition, with the extruder being completely empty. However, during more typical operations where the extruder still contains residual plastic in the hot chamber, a higher temperature is required for purging. As a results, an initial temperature of 220°C was set and maintained until the extrusion speed stabilized at the correct flow rate. Once a consistent extrusion speed was reached, the temperature was lowered to 210°C and kept constant for the remainder of the process. To maintain consistent filament diameter, the hopper must be continuously refilled; otherwise, a reduction in material leads to decreased pushing force in the extruder, causing the filament to become thinner.

**TABLE 2:** Extrusion and spooling parameters used after optimized tuning. In particular, temperatures and speed of extrusion and power of cooling.

Machine	Parameter	Value
Extruder	Temperature [°C]	210
	RPM	9
Spooler	Diameter [mm]	1.70
	Puller Speed [m/min]	0.4
	Traverse Speed [m/min]	4
	Spool Speed [m/min]	30
	Fan Speed	255 (maximum value)

### 3.2 Printing process

**FIGURE 7:**
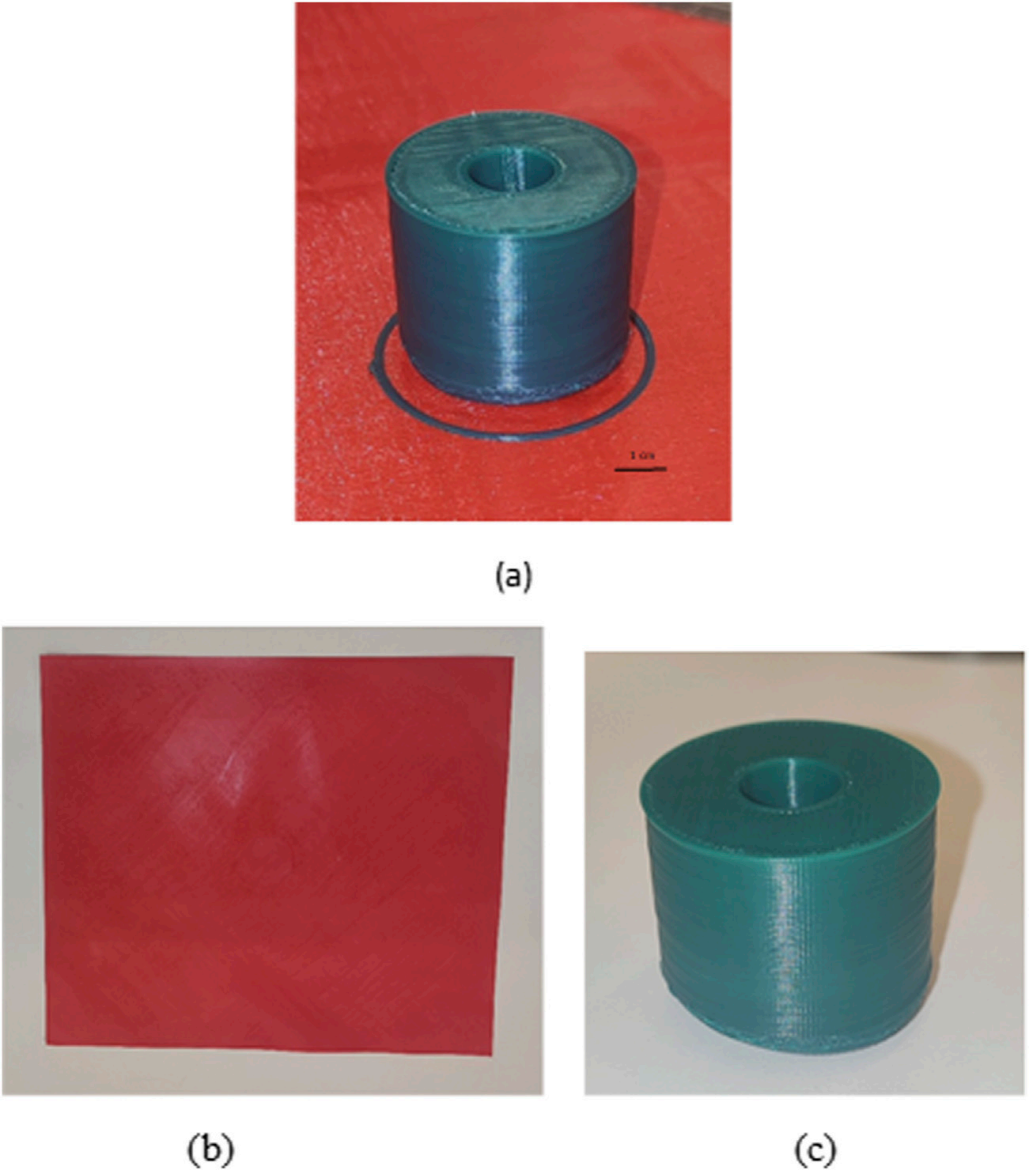
**(A)** HDPE printed object on TPU plate **(B)** TPU plate **(C)** detached HDPE printed object. Slight warping is visible.

The solution identified to overcome issues regarding adhesion and warping during printing was to change the material of the plate, replacing it with a thin layer of thermoplastic polyurethane (TPU) material (Figures 7A, B). Later, a cylinder in HDPE was printed, imposing printing temperature at 260°C, and printing speed 20 mm/s, while the build plate temperature was set at 100°C. Moreover, four rafting layers were added. The resulting object presented good adhesion, easy detachment, and high-quality precision. However, slight warping was identified (Figure 7C).

### 3.3 Mechanical properties

#### 3.3.1 Test 1

**FIGURE 8:**
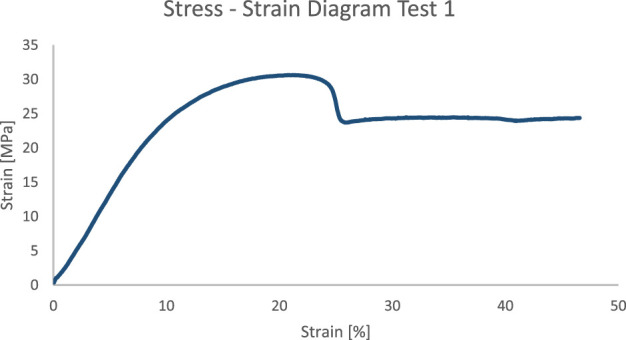
Stress-strain curve test 1.

The stress-strain curve (Figure 8) obtained from the tensile test on the thin filament exhibits typical polymeric behaviour. A slight slippage of the sample can be observed at a strain of 24%. Two key parameters were derived from the graph: Young’s Modulus, which corresponds to the slope of the elastic deformation region (the portion where the deformation remains reversible), and the Tensile Stress at Yielding, which is the stress at which the specimen begins to yield. Although. The test did not reach the fracture point for equipment limitation, yielding was observed within the test length. The Young’s Modulus is approximately 300 MPa, while the Tensile stress at Yielding is 30 MPa.

#### 3.3.2 Test 2

The second test was conducted with the same set-up, but with a thicker filament. The nominal diameter of the filament tested was 1.5mm, which is closer to the diameter typically used in most 3D printers.

**FIGURE 9:**
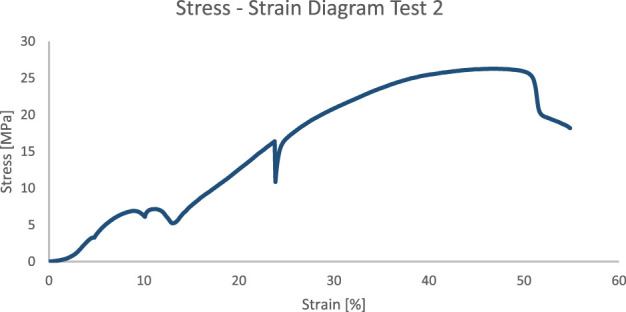
Stress-strain curve test 2.

Several spikes are visible in the stress-strain diagram (Figure 9), indicating instability of the filament predisposition within the set-up.

**FIGURE 10:**
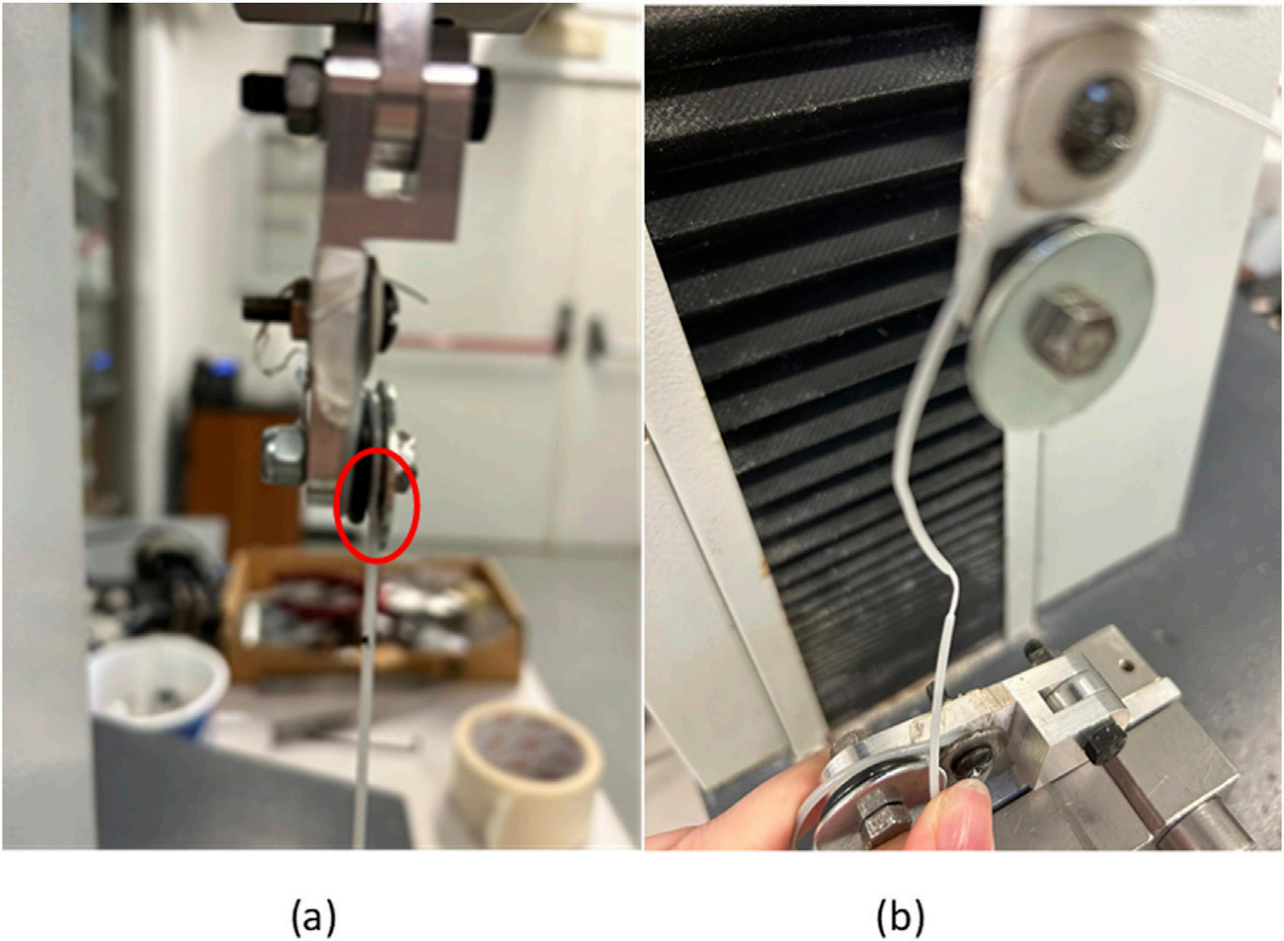
**(A)** Thick filament set-up **(B)** Yielding under clamp, outside of the test length.

Moreover, the yielding of the filament was visible outside the test length, in particular under the flat washer (Figures 10A, B).

#### 3.3.3 Test 3

**FIGURE 11:**
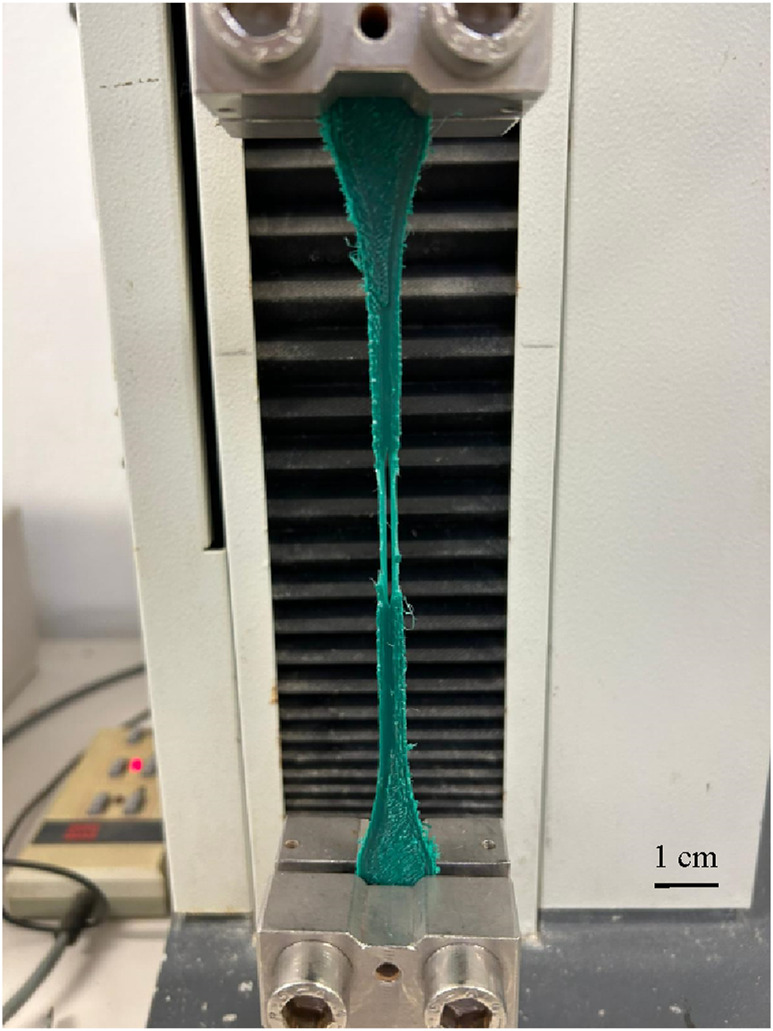
Yielding and cracking Specimen Type II.

In the third test, the specimen began yielding from the external jagged layers at a force significantly lower than anticipated. As the deformation increased, the specimen cracked at the centre, indicating separation of consecutive layers (Figure 11).

**FIGURE 12:**
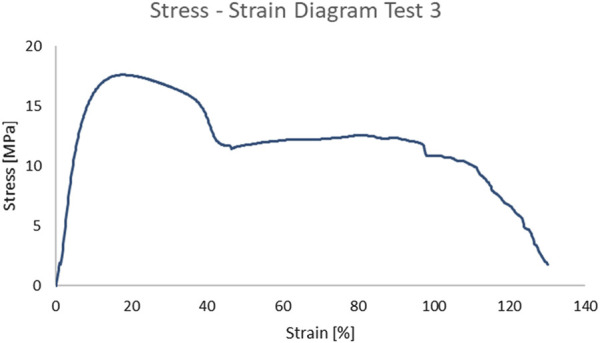
Stress-strain curve Test 3.

The resulting stress-strain curve is irregular, and no definitive conclusions can be drawn from it (Figure 12).

### 3.4 Economic analysis

An economic analysis was performed to evaluate whether in-house production of HDPE filament results in a lower economic impact compared to purchasing industrially produced filament made from the same material. The calculated production costs are summarized in Table 3. The costs were then compared to the average price of commercially available HDPE filament.

**TABLE 3:** Production costs for circular economy process. Equipment costs, energy consumption and man labour.

Costs source	Costs per kg [€]
Equipment costs	0.9
Energy consumption	0.062
Man labour	7
Total Costs	7.962

The prices found were then readjust to reflect the cost per kilogram, and the average price was calculated to be in 34.20€/kg, excluding taxes. Therefore, the difference between in-house production and the purchasing of the filament accounts to 29.24€/kg, representing a cost saving of 85.49%.

### 3.5 Sustainability analysis

Following the energetic consumption data required to produce 1 kg of filament, as presented in the economic analysis, the corresponding CO_2_ emissions were calculated, with the assumption that 1  MW h of energy consumption produces 0.483 tons of CO_2_. The emissions were as follows: the shredder produced 0.145 kg of CO_2_, the extruder 0.206kg, and the spooler 0.121 kg of CO_2_ for 1 kg of HDPE filament.

The last aspect influencing environmental sustainability is transportation, that was considered only for the industrial process. To estimate the CO_2_ emissions from transportation, the same distributors considered in the economic analysis were analysed, along with the distances from these distributors to the Humanitas Research Hospital.

An average CO_2_ emissions rate of 130 gCO_2_/km was used for car transportation, based on historical data showing emissions varying between 120 gCO_2_/km and 140 gCO_2_/km over the last decade. This led to an average transportation emission of 44 kgCO_2_. The results of this analysis are summarized in Table 4. It could be argued that the CO_2_ emission for the production of the bottle caps should be taken into account in the overall emissions of the recycled filament. However, this reasoning does not apply since the fabrication of the bottle caps is independent of its recycling.

**TABLE 4:** CO2 emission comparison. Raw material extraction, material processing and transportation.

	Circular economy process	Industrial process
Raw material extraction	—	3.11kgCO2
Material Processing	0.472kgCO2	9.5kgCO2
Transportation	—	44kgCO2
Total	0.472kgCO2	56.61kgCO2

Based on these calculations, in-house production of HDPE filament results in a 99.16% reduction in CO2 emissions.

### 3.6 Case study

**FIGURE 13:**
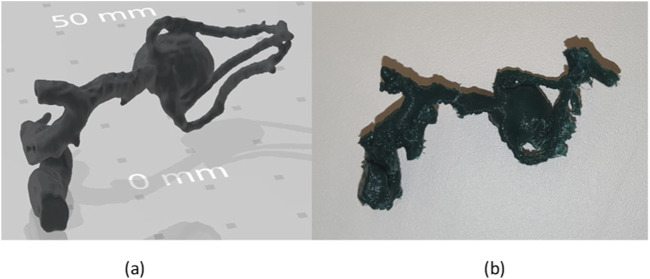
**(A)** 3D model of aneurysm **(B)** 3D printed aneurysm.

The reconstructed and printed model are shown in Figure 13. The impact such models have on the clinical procedure are confirmed by the surgeon, who stated that the possibility to visualize the 3D structure of the aneurysm allowed a more confident pre-operative planning.

## 4 Discussion

The growing prevalence of chronic diseases and the aging population has led to an increase in healthcare waste, which is further exacerbated by the extensive use of plastics in healthcare. Improper disposal of these plastics contributes significantly to environmental harm.

Conversely, the adoption of 3D printing technologies in healthcare is expanding rapidly, with the creation of anatomical models and patient-specific medical devices proving to be valuable tools in clinical practice. In an effort to reduce healthcare waste while fostering innovation through 3D printing, this study develops and implements a circular economy process that recycles plastic bottle caps into 3D printable filament (Aimar et al., 2019).

Mechanical recycling of plastics for 3D printable filament production is a well-established practice. For example, *Mikula et al.* explore the recycling of various plastics, assessing their mechanical properties and degradation (Mikula et al., 2021). However, most studies in this area do not address economic and environmental sustainability—critical factors in the healthcare sector. Typically, research focuses on the design and efficiency of circular economy workflows without real-world application in specific industries (Madhu et al., 2022).

This study, building on existing literature, demonstrates the positive impact of implementing a circular economy process in a crucial sector like healthcare. The results demonstrate the quality of the recycled material and the printability of the filament. Furthermore, an environmental and economic sustainability analysis is performed, showing that process reduces CO_2_ emissions and lowers costs for healthcare institutions. This is particularly for 3D printing facilities in hospitals, where costs management can hinder the widespread adoption of point-of-care 3D printing (Ostas et al., 2022). By reducing material costs through the recycling of plastic waste, this process can facilitate the in-house production of medical devices, which is vital for economic sustainability in healthcare.

Most importantly, the ultimate goal of this work is to enhance patient care. Recent studies have highlighted the positive effects of 3D printing on clinical outcomes and patient trust (Tam et al., 2014; Tevanov et al., 2017). The ability to visualize anatomical models before surgery helps surgeons plan the most effective approach, boosting confidence and reducing surgical time.

Although this study successfully demonstrates the value of a circular economy in the healthcare sector, there is room for improvement. Issues such as printing difficulties could be addressed by incorporating additives into the filament to reduce warping and enhance adhesion (Graziano et al., 2019). Additionally, the workflow could be expanded to include various plastic materials, further minimizing environmental impact.

In conclusion, this research serves as a starting point for healthcare institutions to promote 3D printing facilities that align with available resources while supporting efforts to reduce CO_2_ emissions.

## Data Availability

The raw data supporting the conclusions of this article will be made available by the authors, without undue reservation.
